# Prevalence of colistin resistance in clinical isolates of *Pseudomonas aeruginosa*: a systematic review and meta-analysis

**DOI:** 10.3389/fmicb.2024.1477836

**Published:** 2024-10-09

**Authors:** Negar Narimisa, Abbasali Keshtkar, Leila Dadgar-Zankbar, Narjess Bostanghadiri, Yasaman Rouein Far, Soheila Shahroodian, Abed Zahedi Bialvaei, Shabnam Razavi

**Affiliations:** ^1^Microbial Biotechnology Research Center, Iran University of Medical Sciences, Tehran, Iran; ^2^Department of Microbiology, School of Medicine, Iran University of Medical Sciences, Tehran, Iran; ^3^Department of Disaster and Emergency Health, School of Public Health, Tehran University of Medical Sciences, Tehran, Iran; ^4^Institute for Chemistry and Biology of the Marine Environment (ICBM), School of Mathematics and Science, Carl von Ossietzky Universität Oldenburg Ammerländer Heerstraße, Oldenburg, Germany

**Keywords:** *Pseudomonas aeruginosa*, cystic fibrosis, infection prevention, treatment regimens, public health

## Abstract

**Objective:**

The emergence of resistance to colistin, the last resort for treating severe infections caused by *Pseudomonas aeruginosa*, poses a significant threat to public health. This meta-analysis aimed to investigate the prevalence of colistin resistance in clinical isolates of *P. aeruginosa.*

**Method:**

A comprehensive search of MEDLINE (PubMed), Web of Science, and Scopus databases was conducted to identify relevant articles published until December 2023. Subsequently, a meta-analysis was performed using Stata software to examine the pooled prevalence of colistin resistance and to conduct subgroup analyses.

**Results:**

A total of 619 studies were included in the meta-analysis, revealing a global prevalence of colistin resistance of 1% among all *P. aeruginosa* isolates. Furthermore, cystic fibrosis patients exhibited the highest resistance to colistin, with a prevalence of 7% among the examined diseases.

**Conclusion:**

The increase in colistin resistance in *P. aeruginosa* in recent years from 2% (in the period of 2006–2010) to 5% (in the period of 2020–2023) underscores the need for implementing infection prevention programs, using appropriate treatment regimens, and disseminating comprehensive information on antimicrobial resistance patterns. These measures are crucial for addressing this growing public health concern.

## Introduction

*Pseudomonas aeruginosa* is recognized as an opportunistic pathogen and a major causative agent of hospital-acquired infections, including urinary tract infections, pneumonia, bloodstream infections, and surgical site infections (Pang et al., 2019; Sadikot et al., 2005). The development of intrinsic and acquired resistance in *P. aeruginosa* is attributed to the inappropriate and excessive use of antibiotics, leading to the emergence of antibiotic resistance (El-Mokhtar and Hetta, 2018).

The management of *P. aeruginosa* infections has always presented challenges. Carbapenems such as imipenem and meropenem were introduced as effective treatments for severe multidrug-resistant (MDR) *P. aeruginosa* infections. However, the overuse of antibiotics has resulted in the emergence of carbapenem-resistant isolates, posing a significant concern (Wi et al., 2017; Bonyadi et al., 2022). In 2017, the World Health Organization (WHO) identified carbapenem-resistant *P. aeruginosa* as a priority pathogen necessitating the development of new antibiotics for treatment (Tacconelli et al., 2018).

The increasing rate of infections caused by multidrug-resistant (MDR), extensively drug-resistant (XDR), and particularly carbapenem-resistant *P. aeruginosa* has led to the resurgence of colistin as a critical last-resort therapeutic option (Wi et al., 2017; Biswas et al., 2012; Al-Orphaly et al., 2021). Despite its potent antimicrobial activity against *P. aeruginosa* and its designation as a potentially effective drug, the increased utilization of colistin has resulted in the emergence of bacterial strains with reduced susceptibility to this antibiotic class worldwide (Pechorsky et al., 2009; Lee et al., 2012).

Colistin resistance primarily arises through various mechanisms, including enzymatic modification of lipid A, leading to a decrease in the outer membrane’s negative charge and reduced colistin affinity. Resistance to colistin may also stem from chromosomally encoded mutations or plasmid-mediated colistin resistance gene *mcr*, facilitating horizontal dissemination of resistance (Cannatelli et al., 2013; Paterson and Harris, 2016; Hasman et al., 2015; Arcilla et al., 2016). The prevalence of colistin resistance is typically below 10%, but this rate is steadily increasing in the Mediterranean, Southeast Asia, and certain African countries (Bialvaei and Samadi, 2015). Recent observations indicate that resistance to colistin has emerged in several Enterobacteriaceae species, including *Klebsiella pneumoniae*, *Escherichia coli*, and *Enterobacter aerogenes*. This resistance has been linked to the extensive use of polymyxins for infection control in veterinary medicine (Baron et al., 2016; Al-Kadmy et al., 2020). Given the potential for both horizontal transfer of resistance genes through conjugative plasmids and vertical transfer through chromosomal mutation, the emergence of colistin-resistant isolates poses a significant global health threat, especially considering the importance of colistin as a last-resort treatment option (Liu et al., 2016; Abd El-Baky et al., 2020).

The rise of MDR, XDR, and pan drug-resistant (PDR) *P. aeruginosa* poses a significant public health challenge, leading to delays in antimicrobial therapy, treatment failures, and increased mortality rates (Abd El-Baky et al., 2020). This situation necessitates urgent attention, as these resistant strains may exhibit resistance to all available antimicrobials or show susceptibility only to colistin or polymyxins, severely limiting treatment options for healthcare providers managing severe infections associated with MDR *P. aeruginosa*. The emergence of colistin-resistant strains is particularly concerning for patients with critical infections. Consequently, this systematic review and meta-analysis aims to investigate the global prevalence of colistin resistance in *P. aeruginosa*, thereby enhancing our understanding of antibiotic resistance in this pathogen.

## Methods

### Search strategy

We conducted a comprehensive search for eligible studies published from 1990 to December 2023 using MEDLINE (PubMed), Web of Science, and Scopus. The search terms included (“*Pseudomonas aeruginosa*” OR *P. aeruginosa*) AND (Colisticin OR “Polymyxin E” OR Colimycin OR colistin OR colistimethate). This review was carried out and reported in accordance with current guidelines, and the results were reported following the Preferred Reporting Items for Systematic Reviews and Meta-Analyses statement (Moher et al., 2009).

### Inclusion and exclusion criteria

All original articles that provided data on the total number of clinical *P. aeruginosa* isolates and the number of colistin-resistant *P. aeruginosa* isolates were included. Studies were excluded if they met the following criteria: (Pang et al., 2019) did not present *P. aeruginosa* colistin resistance; (Sadikot et al., 2005) did not clearly report resistance rates (the exact number of primary isolates and the number of resistant isolates are not provided); (El-Mokhtar and Hetta, 2018) conducted antimicrobial susceptibility tests for colistin without specifying the method; (Wi et al., 2017) were written in languages other than English; and (Bonyadi et al., 2022) sourced data from conference abstracts, editorials, case reports, meta-analyses, systematic reviews, narrative reviews, experimental studies on animal models, and articles without full text after contacting the corresponding author.

### Data extraction

After consolidating the articles using EndNote X20 Citation Manager Software, duplicate articles were removed before review. The citations were then imported into Rayyan, a citation classification application (Ouzzani et al., 2016). Three reviewers independently screened all titles and abstracts to exclude irrelevant topics. In the subsequent assessment stage, qualified studies were downloaded, and the full text of selected articles was retrieved based on the inclusion and exclusion criteria.

Three reviewers developed a data extraction form and collected data from all qualified studies. The extracted data included the first author’s name, year of publication, year of collection, continent and countries where the study was conducted, sample size (number of *P. aeruginosa* isolates and number of colistin-resistant isolates), origin of isolates, drug resistance categories, disease, guideline, and susceptibility test methodology (agar dilution, broth microdilution, disk elution, E-test, and disk diffusion).

### Quality assessment

The quality of the included studies was evaluated independently by three reviewers using a modified version of the Joanna Briggs Institute (JBI) assessment tool for prevalence studies (Munn et al., 2014). The checklist includes the following questions: Was the sample frame appropriate to address the target population? Were study participants sampled appropriately? Was the sample size adequate? Were the study subjects and the setting described in detail? Was the data analysis conducted with sufficient coverage of the identified sample? Were valid methods used for the identification of the condition? Was the condition measured in a standard, reliable way for all participants? Was there an appropriate statistical analysis? Was the response rate adequate, and if not, was the low response rate managed appropriately? Each item is evaluated as “yes,” “no,” or “unclear.” A “yes” response is assigned a score of 1 point, while responses categorized as “no” or “unclear” receive 0 points. Studies that score 7 or higher are classified as high quality, those with scores between 5 and 6 are considered medium quality, and studies scoring 4 or lower are designated as low quality. In cases of disagreement, a fourth reviewer provided adjudication.

### Statistical analysis

We conducted a prevalence meta-analysis using the metaprop package in Stata 17 software. We calculated the pooled prevalence of colistin-resistant *P. aeruginosa*, along with the associated 95% confidence intervals (CIs), utilizing the Freeman-Tukey double arcsine transformation within a random-effects model.

To identify publication bias, we employed Egger’s test, with a significance threshold set at *p* < 0.05, indicating the presence of statistically significant bias. Additionally, a trim-and-fill analysis was conducted to address potential bias. Funnel plots were also utilized for a visual assessment of publication bias.

Heterogeneity among studies was measured using the I^2^ statistic. Specifically, I^2^ ≤ 25% indicated low heterogeneity, 25% < I^2^ ≤ 75% indicated moderate heterogeneity, and I^2^ > 75% indicated high heterogeneity.

Subgroup analyses were conducted based on various factors, including publication year (from 2009 to 2023), collection period (five distinct periods), continent (five continents), country (thirty-two countries), guidelines followed (CLSI and EUCAST), disease type (including Urinary Tract Infections, Pneumonia, Lower Respiratory Tract Infections, Intra-abdominal Infection, COVID-19, cancer, Cystic Fibrosis, Bacteremia, and Bloodstream Infection), method of colistin resistance detection (agar dilution, E-test, disk diffusion, and broth microdilution), different resistance categories (Multidrug Resistance, Extensively Drug Resistant, and Carbapenem Resistance), and sample origin (urine, sputum, endotracheal aspirate, burn wounds, and blood).

## Results

### Studies selection

Our initial search yielded a total of 9,378 articles. After removing duplicates, we screened the titles and abstracts of 7,561 articles. From this screening process, 1,076 articles met the inclusion criteria and were selected for a full-text review. After the full-text review, we identified 619 articles that were suitable for analysis (Abavisani et al., 2021; Abd El-Baky et al., 2020; Abdelatti et al., 2023; Abed and Kareem, 2021; Abubakar et al., 2022; Abulzahra and Ismail, 2020; Addis et al., 2021; Agrawal et al., 2013; Aguilar-Rodea et al., 2017; Ahani Azari and Fozouni, 2020; Ahmed et al., 2022; Ahmed et al., 2019; Aiyegoro et al., 2007; Akgül et al., 2021; Akhi et al., 2015; Akram et al., 2022; Al Dawodeyah et al., 2018; Al-Agamy et al., 2012; Al-Bayssari et al., 2021; Al-Kabsi et al., 2011; Al-kaffas et al., 2022; Al-Khudhairy and Al-Shammari, 2020; Al-shimmary, 2018; Al-Zahrani and Al-Ahmadi, 2021; Alam et al., 2021; Alcántar-Curiel et al., 2023; Alexopoulou et al., 2016; Alfouzan et al., 2022; Alfouzan et al., 2018; Alhanout et al., 2009; Ali et al., 2021; Ali et al., 2015; Alkhulaifi and Mohammed, 2023; Alotaibi et al., 2023; Alruways, 2023; Amabile-Cuevas, 2017; Appalaraju et al., 2020; Aprile et al., 2019; Arab et al., 2023; Araújo Lima et al., 2020; Arca-Suárez et al., 2021; Arici et al., 2023; Arif et al., 2022; Armengol et al., 2020; Armengol et al., 2019; Aruhomukama et al., 2019; Asar et al., 2019; Aydemir et al., 2022; Aydın et al., 2018; Azimi et al., 2012; Azimi L. et al., 2016; Azimi S. et al., 2016; Babu and Menon, 2018; Badawy et al., 2023; Badierah et al., 2019; Bae and Stone, 2022; Baek et al., 2020; Bagheri-Nesami et al., 2017; Bahabri et al., 2022; Bahador et al., 2019; Bahçe et al., 2022; Baiomy et al., 2023; Bakht et al., 2022; Balkhair et al., 2023; Bandic-Pavlovic et al., 2020; Banerjee et al., 2024; Bangera et al., 2016; Basu et al., 2013; Bayram et al., 2013; Bazgir et al., 2021; Beirao et al., 2020; Ben Nejma et al., 2018; Berwal et al., 2020; Bian et al., 2022; Blondeau et al., 2023; Bogiel et al., 2022; Bono et al., 2015; Bourgi et al., 2020; Boustanshenas et al., 2023; Brauncajs et al., 2022; Brzozowski et al., 2020; Bunsow et al., 2020; Buzilă et al., 2021; Cabot et al., 2011; Camargo et al., 2023; Candel et al., 2022; Canton et al., 2022; Carvalhaes et al., 2020; Castanheira et al., 2018; Cavallo et al., 2022; Cesur et al., 2012; Çetin et al., 2022; Chang et al., 2023; Chaturvedi et al., 2021; Chaudhary et al., 2020; Chauhan et al., 2022; Chen et al., 2022; Chen et al., 2015; Chen et al., 2023; Chen Q. et al., 2020; Chen X. et al., 2020; Chen et al., 2014; Chew et al., 2019; Chittawatanarat et al., 2014; Chukamnerd et al., 2023; Cillóniz et al., 2016; Cipriano et al., 2007; Çopur Çiçek et al., 2021; Czekajło-Kołodziej et al., 2006; da Costa Júnior et al., 2020; Dadmanesh et al., 2014; Darji and Patel, 2023; Dassner et al., 2017; Datar et al., 2021; de Dios et al., 2016; De Francesco et al., 2013; de Oliveira Santos et al., 2019; De Vecchi et al., 2013; Dehbashi et al., 2018; Del Barrio-Tofino et al., 2017; Del Giacomo et al., 2022; Delgado-Valverde et al., 2020; Delroshan et al., 2023; Depka et al., 2020; Descours et al., 2018; Dharati et al., 2021; Di Carlo et al., 2021; Di Domenico et al., 2017; Dias et al., 2017; Díaz-Cañestro et al., 2018; Diekema et al., 2019; Din et al., 2023; Do Tran et al., 2022; Dogonchi et al., 2018; Dong et al., 2012; Doumith et al., 2022; Durdu et al., 2018; Ebadati et al., 2023; Ece et al., 2014; Eftekhar et al., 2009; Eid et al., 2020; Ejaz, 2022; Ekkelenkamp et al., 2020; El Mekes et al., 2020; El-Sokkary et al., 2021; Eladawy et al., 2021; Elshafie et al., 2007; Emami et al., 2017; Emami et al., 2019; Emami et al., 2020; Ergul et al., 2017a; Ergul et al., 2017b; Escolà-Vergé et al., 2018; Evans et al., 2019; Fakhkhari et al., 2022; Falagas et al., 2017; Fang et al., 2023; Farag et al., 2020; Farhan et al., 2019; Farrell et al., 2013; Farrell et al., 2014a; Farrell et al., 2014b; Fekri Kohan et al., 2020; Feretzakis et al., 2019; Ferjani et al., 2022; Fernández-Olmos et al., 2013; Flamm et al., 2014; Flores-Paredes et al., 2021; Fluge et al., 2001; Ford et al., 2022; Fournier et al., 2021; Fournier et al., 2013; Fraenkel et al., 2023; Franco et al., 2010; Franco et al., 2023; Gaber et al., 2020; Gahlot and Kasana, 2021; Gajdács et al., 2021a; Gajdács et al., 2019; Gajdács et al., 2021b; Galani et al., 2008; Galani et al., 2020; Gales et al., 2012; Gales et al., 2011; Gales et al., 2003; Galindo-Mendez et al., 2023; Gangwar et al., 2021; Gant et al., 2021; García-Castillo et al., 2011; Garcia-Fernandez et al., 2019; Garg et al., 2019; Gaudereto et al., 2020; Ghanem et al., 2023; Ghasemian et al., 2023; Ghasemshahi et al., 2022; Gherardi et al., 2019; Giani et al., 2018; Gilani et al., 2015; Goli et al., 2017; Goli et al., 2016; Golli et al., 2022; Gomez et al., 2023; Gómez-Garcés et al., 2009; Gomila et al., 2013; Gunalan et al., 2021; Guo et al., 2023; Gutierrez-Santana et al., 2022; Guzek et al., 2017; Guzek et al., 2015; Güzel and Gerçeker, 2008; Hackel et al., 2018; Hakeam et al., 2022; Hallit et al., 2020; Hansen et al., 2008; Hao et al., 2023; Hawser et al., 2021; Heidari et al., 2022; Henderson et al., 2018; Herrera-Espejo et al., 2020; Hindler and Humphries, 2013; Hishinuma et al., 2018; Hishinuma et al., 2020; Holger et al., 2023; Holger et al., 2022a; Hong et al., 2015; Hoşbul et al., 2022; Hosseininassab Nodoushan et al., 2017; Howard-Anderson et al., 2022; Hrbacek et al., 2020; Hsueh et al., 2019; Hu et al., 2022; Hu et al., 2023; Hu et al., 2021a; Hu et al., 2021b; Huang et al., 2022; Huband et al., 2016; Huband et al., 2020; Humphries et al., 2023; Ibrahim, 2018; Ioannou et al., 2023; Ismail and Mahmoud, 2018; Izadi Pour Jahromi et al., 2018; Jalali et al., 2021; Jamalifar et al., 2019; Jansen et al., 2016; Japoni et al., 2011; Jauhari et al., 2020; Jayarani et al., 2020; Jazani et al., 2012; Jean et al., 2009; Jean et al., 2016; Jean et al., 2022; Jeong et al., 2024; Jimenez-Guerra et al., 2018; Jones et al., 2013a; Jones et al., 2014; Jones et al., 2013b; Juhász et al., 2017a; Juhasz et al., 2017b; Jung et al., 2019; Kabic et al., 2023; Kakhandki et al., 2020; Kanwar et al., 2023; Karami et al., 2020; Karimzadeh et al., 2017; Karlowsky et al., 2013; Karlowsky et al., 2022; Karlowsky et al., 2019a; Karlowsky et al., 2019b; Karlowsky et al., 2023; Karlowsky et al., 2018a; Karlowsky et al., 2018b; Karlowsky et al., 2021; Karruli et al., 2022; Kashfi et al., 2017; Katchanov et al., 2018; Kaur et al., 2022; Kazmierczak et al., 2018; Kazmierczak et al., 2016; Kc et al., 2019; Keepers et al., 2014; Khajuria et al., 2013; Khan et al., 2017; Khan et al., 2023; Khater, 2022; Khater and AlFaki, 2022; Khorvash et al., 2017; Khoshnood et al., 2019; Khosravi et al., 2017; Khosravi et al., 2016; Kidd et al., 2009; Kim et al., 2022; Kim et al., 2017; Kiratisin et al., 2021; Kiratisin et al., 2023; Kırac et al., 2018; Ko and Stone, 2020; Ko and Stone, 2020; Kohira et al., 2023; Kokkayil et al., 2018; Kombade et al., 2021; Korzekwa et al., 2021; Kothari et al., 2022; Kovacevic et al., 2019; Kragh et al., 2021; Krause et al., 2019; Kresken et al., 2020a; Kresken et al., 2020b; Kristof et al., 2021; Kuo et al., 2021; Kurihara et al., 2022; Lai et al., 2019; Laurentiu et al., 2017; Lee J.-Y. et al., 2011; Lee S.-C. et al., 2011; Lee et al., 2023; Lee et al., 2019; Li et al., 2021; Li et al., 2022; Li et al., 2023; Licata et al., 2020; Lin J. et al., 2019; Lin et al., 2016; Lin Z. et al., 2019; Liu et al., 2018; Liu et al., 2020; Livermore et al., 2017; Llanes et al., 2013; Lob et al., 2023; Lockwood and Lawson, 1973; Longshaw et al., 2020; López Montesinos et al., 2022; Lopez-Causape et al., 2017; Lopez-Causape et al., 2013; Macin and Akyon, 2017; MacKenzie et al., 2004; Mahar et al., 2020; Mahdy, 2022; Mahmoud et al., 2021; Maina et al., 2023; Makharita et al., 2020; Malekzadegan et al., 2019; Mallikarjuna and Dhanashree, 2023; Manno et al., 2005; Mansour et al., 2013; Maraki et al., 2015; Maraki et al., 2012; Marteva-Proevska et al., 2021; Martínez et al., 2012; Mataraci Kara et al., 2020; Matuschek et al., 2018; McCracken et al., 2019; McCracken et al., 2019; Medell et al., 2013; Medell et al., 2012; Mellouli et al., 2021; Memar et al., 2016; Mendes et al., 2013; Meradji et al., 2016; Meradji et al., 2015; Meschiari et al., 2021; Mickymaray, 2019; Mikucionyte et al., 2016; Milczewska et al., 2020; Milojković et al., 2020; Mirbagheri et al., 2015; Mishra et al., 2020; Mobaraki et al., 2018; Mohamed et al., 2021; Mohamed et al., 2022; Mohamed and Youssef, 2011; Mohammadi Barzelighi et al., 2020; Mohammadzadeh et al., 2017; Mohammadzamani et al., 2020; Mohanty et al., 2013; Momenah et al., 2023; Monogue and Nicolau, 2018; Montero M. et al., 2020; Montero et al., 2019; Montero M. M. et al., 2020; Moradi et al., 2021; Morata et al., 2012; Mordi and Erah, 2006; MaI et al., 2005; Morrow et al., 2013; Morteza et al., 2019; Mostafa et al., 2022; Moubareck et al., 2019; Mulet et al., 2021; Mustafa et al., 2016; Naas et al., 2021; Nair et al., 2020; Najafi et al., 2015; Nedeljković et al., 2015; Negm et al., 2023; Negm et al., 2021; Nelson and Rosowsky, 2002; Nguyen et al., 2021; Nichols et al., 2016; Nitescu et al., 2023; Nitz et al., 2021; Nojookambari et al., 2019; Nolan et al., 2021; Nwabuisi and Ologe, 2002; O'Carroll et al., 2004; Odumosu et al., 2012; Olowo-okere et al., 2020; Öncül et al., 2014; Ozumba, 2005; Pani et al., 2022; Papagiannitsis et al., 2017; Paprocka et al., 2021; Park et al., 2017; Parsa et al., 2020; Pasca et al., 2012; Peña et al., 2012; Pérez et al., 2019; Perez et al., 2014; Pérez-Vázquez et al., 2020; Perovic et al., 2023; Petca, 2021; Petca, 2021; Petrova et al., 2016; Peymani et al., 2015; Pfaller et al., 2022; Pfaller et al., 2017; Pfaller et al., 2018; Picão Renata et al., 2009; Pierard and Stone, 2021; Pitt et al., 2003; Pourakbari et al., 2016; Pragsam et al., 2018; Prasanth Manohar et al., 2017; Kokkayil et al., 2018; Priyadarshi et al., 2023; Pujji et al., 2019; Qadeer et al., 2016; Radan et al., 2016; Rafalskiy et al., 2020; Rahimi et al., 2021; Rajenderan et al., 2014; Ramadan et al., 2018; Ramanathan et al., 2017; Rashid et al., 2014; Rattanaumpawan et al., 2013; Raza et al., 2019; Reddy et al., 2014; Rizek et al., 2014; Rodulfo et al., 2019; Rolston et al., 2020; Rosales-Reyes et al., 2020; Rossolini et al., 2008; Rout et al., 2023; Roy Chowdhury et al., 2016; Ruekit et al., 2022; Ruh et al., 2016; Ruiz-Roldan et al., 2018; Sacha et al., 2015; Sader et al., 2020a; Sader et al., 2020b; Sader et al., 2019; Sader et al., 2018a; Sader et al., 2015a; Sader et al., 2017a; Sader et al., 2016; Sader et al., 2017b; Sader et al., 2017c; Sader et al., 2018b; Sader et al., 2017d; Sader et al., 2021a; Sader et al., 2014a; Sader et al., 2014b; Sader et al., 2014c; Sader et al., 2018c; Sader et al., 2018d; Sader et al., 2018e; Sader et al., 2017e; Sader et al., 2015b; Sader et al., 2021b; Saderi et al., 2015; Saffari et al., 2017; Saleem M. et al., 2023; Saleem et al., 2022; Saleem and Bokhari, 2020; Saleem Z. et al., 2023; Salimizand et al., 2023; Samonis et al., 2012; Samonis et al., 2010; Samonis et al., 2012; Sanchez-Lopez et al., 2021; Santella et al., 2021; Santimaleeworagun et al., 2020; Sarwat et al., 2021; Schaumburg et al., 2022; Schechner et al., 2011; Schülin, 2002; Seale et al., 1979; Sefraoui et al., 2014; Seifert et al., 2018; Şen et al., 2016; Sendra et al., 2022; Shahri et al., 2022; Sharan et al., 2016; Shariati et al., 2018; Sharifi et al., 2019; Sharma et al., 2023; Sherchan et al., 2022; Sherchan and Humagain, 2020; Sheth et al., 2012a; Sheth et al., 2012b; Shiralizadeh et al., 2023; Shivshetty et al., 2020; Shortridge et al., 2021a; Shortridge et al., 2021b; Shortridge et al., 2017; Shortridge et al., 2019a; Shortridge et al., 2019b; Shortridge et al., 2019c; Shortridge et al., 2018; Shortridge et al., 2020; Shortridge et al., 2022; Shravani et al., 2023; Sid Ahmed et al., 2020; Sid Ahmed et al., 2019; Sid Ahmed et al., 2021; Sid Ahmed et al., 2021; Sid Ahmed et al., 2023; Simar et al., 2017; Singh-Moodley et al., 2018; Singhal et al., 2019; Sleiman et al., 2023; Soliman et al., 2020; Soni et al., 2023; Stone and Ponce-de-Leon, 2020; Stone et al., 2020; Stracquadanio et al., 2021; Tada et al., 2019; Tada et al., 2013; Tahmasebi et al., 2020a; Tahmasebi et al., 2020b; Tahmasebi et al., 2020c; Takahashi et al., 2021; Taleb et al., 2023; Talebi and Hakemi-Vala, 2019; Tan and Ng, 2006; Tantisiriwat et al., 2022; Tarashi et al., 2016; Taylor et al., 2021; Tchakal-Mesbahi et al., 2021; Tekin et al., 2013; Tenover et al., 2022; Thabet et al., 2022; Thapa et al., 2023; Thelen et al., 2022; Tiengrim et al., 2017; Tohamy et al., 2018; Tsitsopoulos et al., 2016; Tumbarello et al., 2013; Tumbarello et al., 2011; Tuon et al., 2020; Khudair and Mahmood, 2021; Ullah et al., 2019; Ullah et al., 2023; Uskudar Guclu et al., 2021; Uzun et al., 2014; Valenza et al., 2010; Valenza et al., 2008; Valenza et al., 2015; Vamsi et al., 2023; Van An et al., 2023; Van An et al., 2023; van Burgh et al., 2019; van der Heijden et al., 2007; Vasudeva et al., 2016; Vata et al., 2018; Vata et al., 2018; Veeraraghavan et al., 2018; Viasus et al., 2020; Viedma et al., 2012; Vosahlikova et al., 2007; Wadhwa et al., 2016; Walkty et al., 2012; Walkty et al., 2011; Walkty et al., 2009; Walkty et al., 2013; Walkty et al., 2022; Walkty et al., 2017; Walters et al., 2019; Wang et al., 2020; Wang and Wang, 2020; Wattal et al., 2019; Wattal et al., 2014; Wemambu and Joshi, 1983; Wendel et al., 2022; Wi et al., 2017; Wi et al., 2018; Willmann et al., 2013; Wise et al., 2023a; Wise et al., 2023b; Wu et al., 2022; Xi et al., 2022; Yadav et al., 2020; Yayan et al., 2015; Yilmaz et al., 2023; Yilmaz et al., 2017; Yousefi et al., 2013; Zerouali et al., 2016; Zhanel et al., 2019; Zhanel et al., 2013; Zhanel et al., 2011; Zhanel et al., 2010; Zhang et al., 2021; Zhao et al., 2023; Zhu et al., 2021; Zhu et al., 2023; Zorgani et al., 2015; Zubair and Iregbu, 2018; Farzana et al., 2013).

A total of 262 articles investigated colistin resistance in *P. aeruginosa* using the microbroth dilution method, while 242 articles employed methods other than microbroth dilution. Additionally, 115 articles examined colistin resistance in multidrug-resistant (MDR), extensively drug-resistant (XDR), and carbapenem-resistant (CR) bacteria.

**Figure 1:**
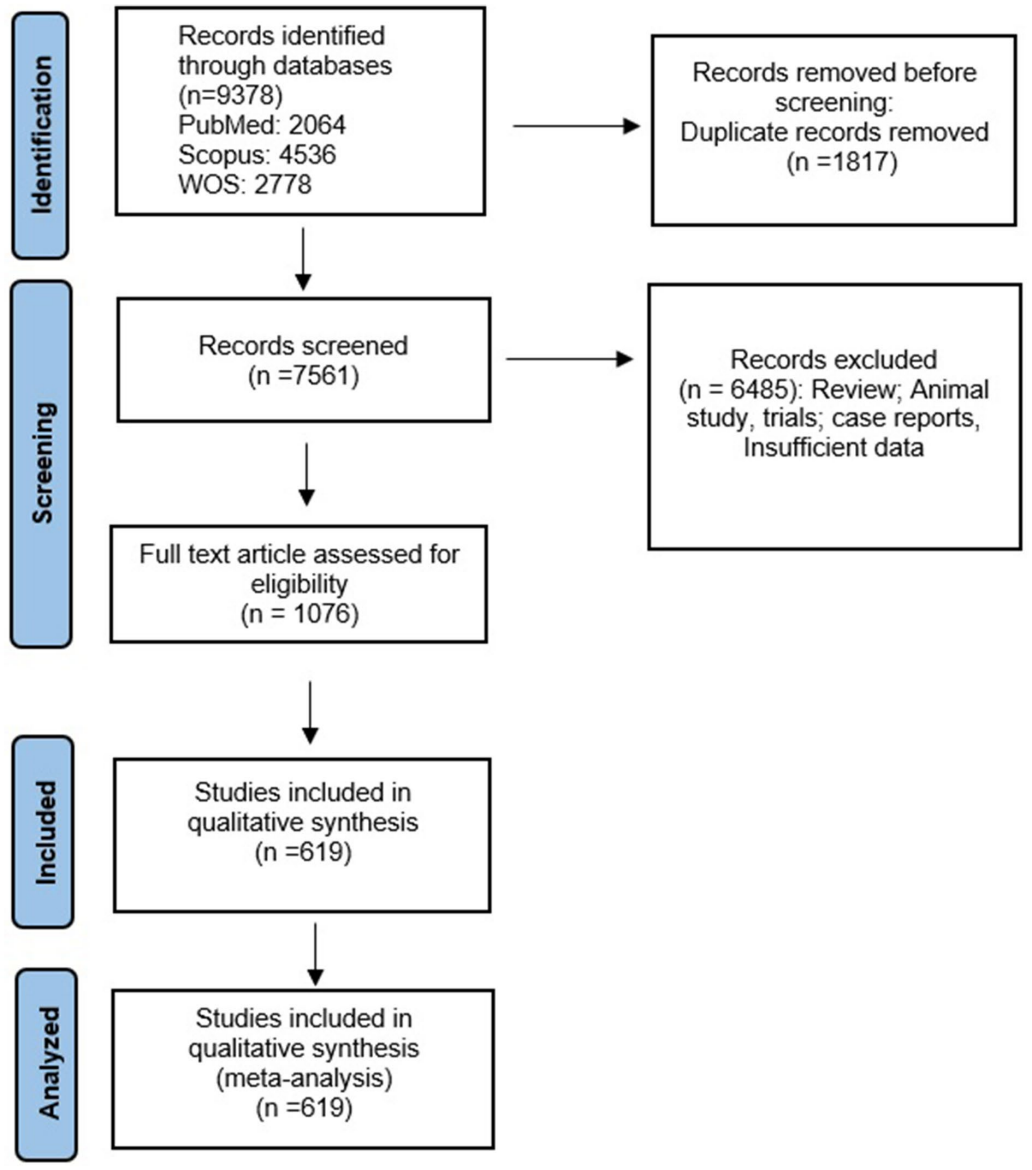
The study PRISMA flow diagram.

We followed the PRISMA guidelines and presented the article selection process in a flow diagram (Figure 1). Supplementary Table S1 provides a summary of the characteristics and quality assessment of all included studies. Additionally, references to the included studies can be found in Supplementary File 2.

### Meta-analysis results

For our meta-analysis, we focused on studies that utilized standard methods such as the broth microdilution and disk elution, which is recommended by the Clinical and Laboratory Standards Institute (CLSI) and the European Committee on Antimicrobial Susceptibility Testing (EUCAST) guidelines for evaluating resistance rates. Out of the total articles reviewed, 262 articles investigated colistin resistance in *P. aeruginosa* isolates using the microbroth dilution method. The pooled prevalence of colistin resistance among clinical *P. aeruginosa* isolates was estimated to be 1% (95% CI: 1–2%; I^2^ = 97.47%; *p* < 0.001).

**Figure 2:**
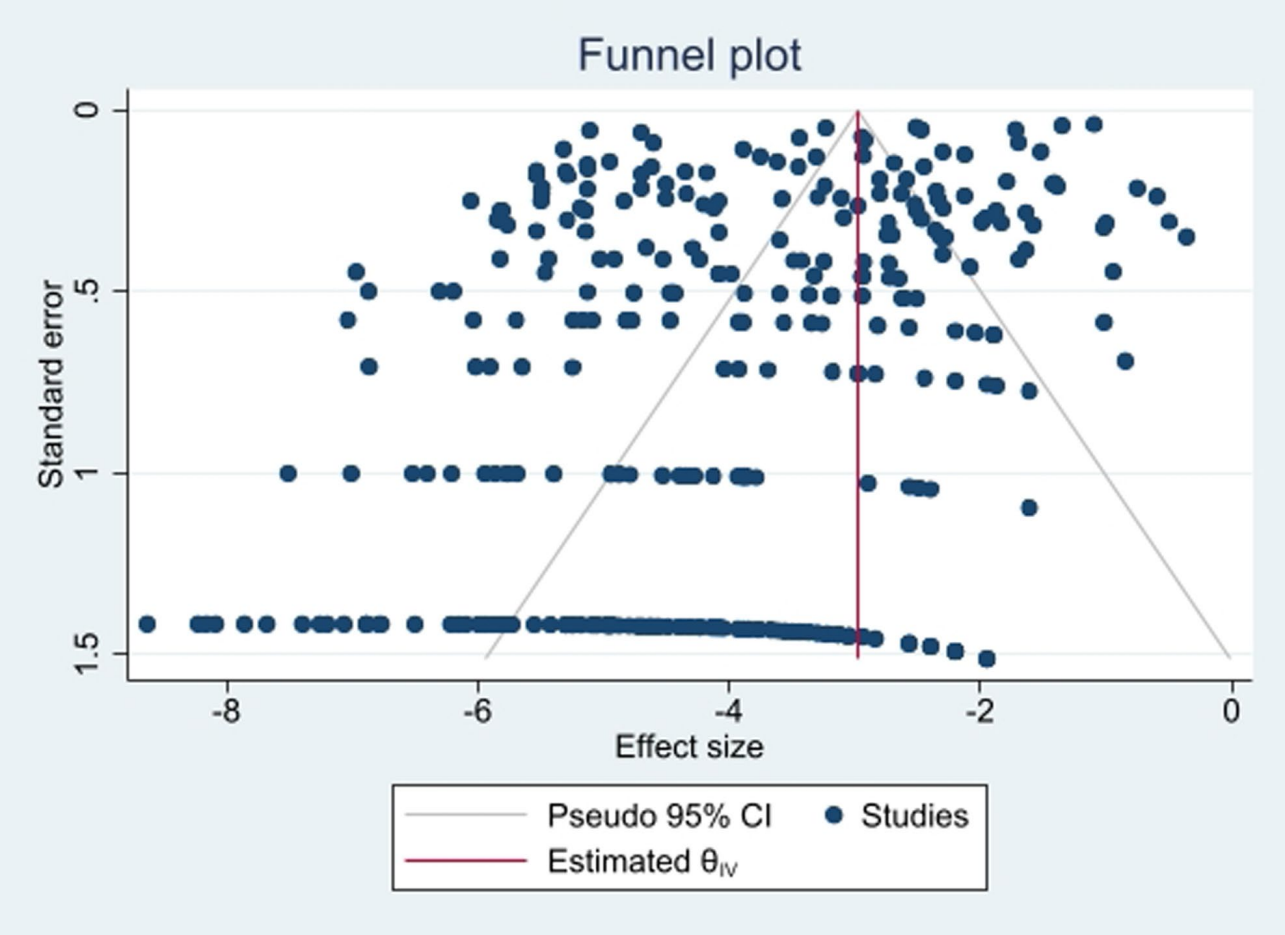
Funnel plot of the prevalence of colistin-resistant *P. aeruginosa* isolates.

To assess publication bias, we examined a funnel plot (Figure 2) and conducted Egger’s tests, which yielded a *p*-value of 0.053, indicating no evidence of publication bias. Additionally, the results following the Trim-and-Fill adjustment indicated that the prevalence of colistin resistance remained unchanged.

### Subgroup meta-analysis

Subgroup meta-analyses were conducted based on various factors, including the year of publication, period of sample collection, continent, country, guideline used, disease assessed, origin of samples, different resistance categories, and methods.

**Figure 3:**
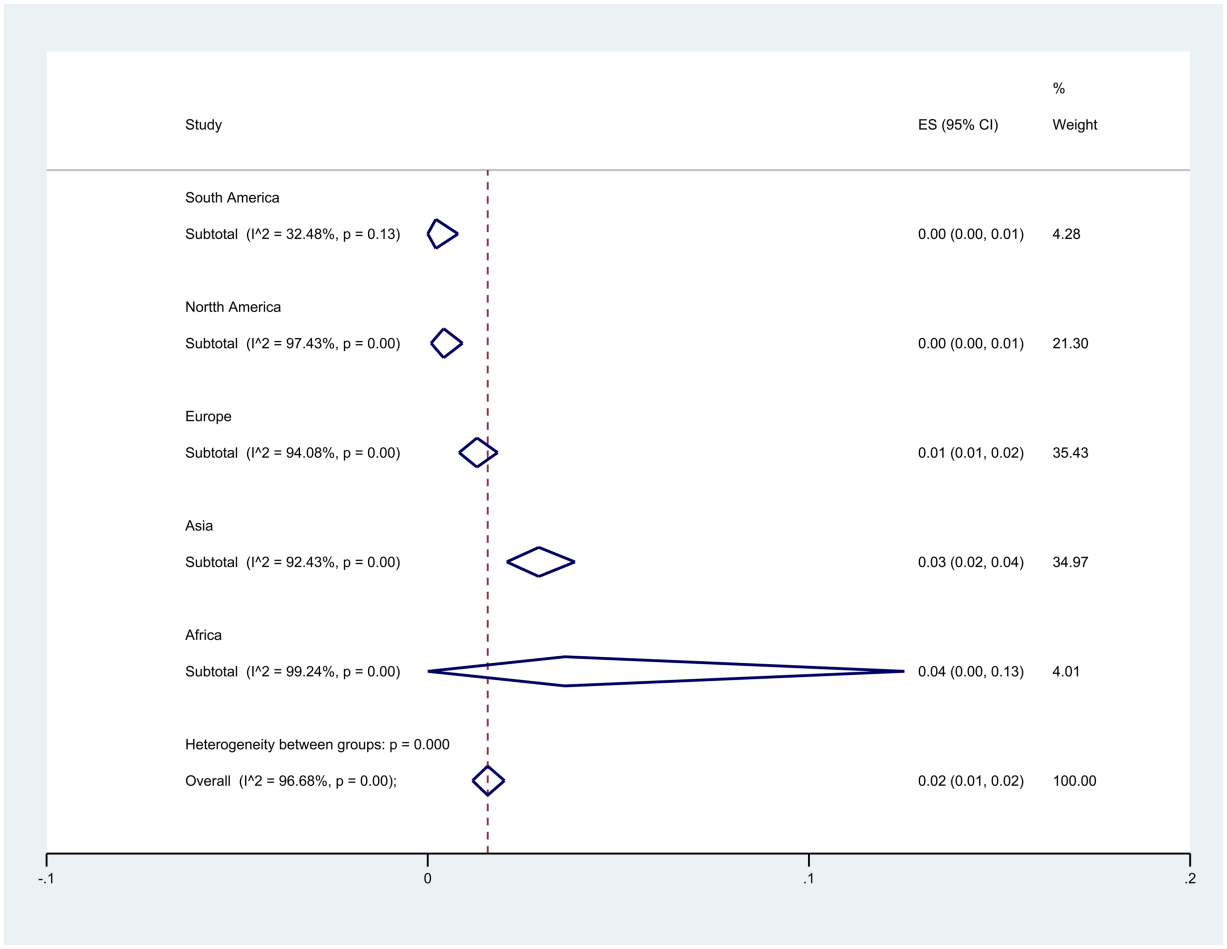
Subgroup analysis for continents of colistin-resistant *P. aeruginosa* isolates.

**Figure 4:**
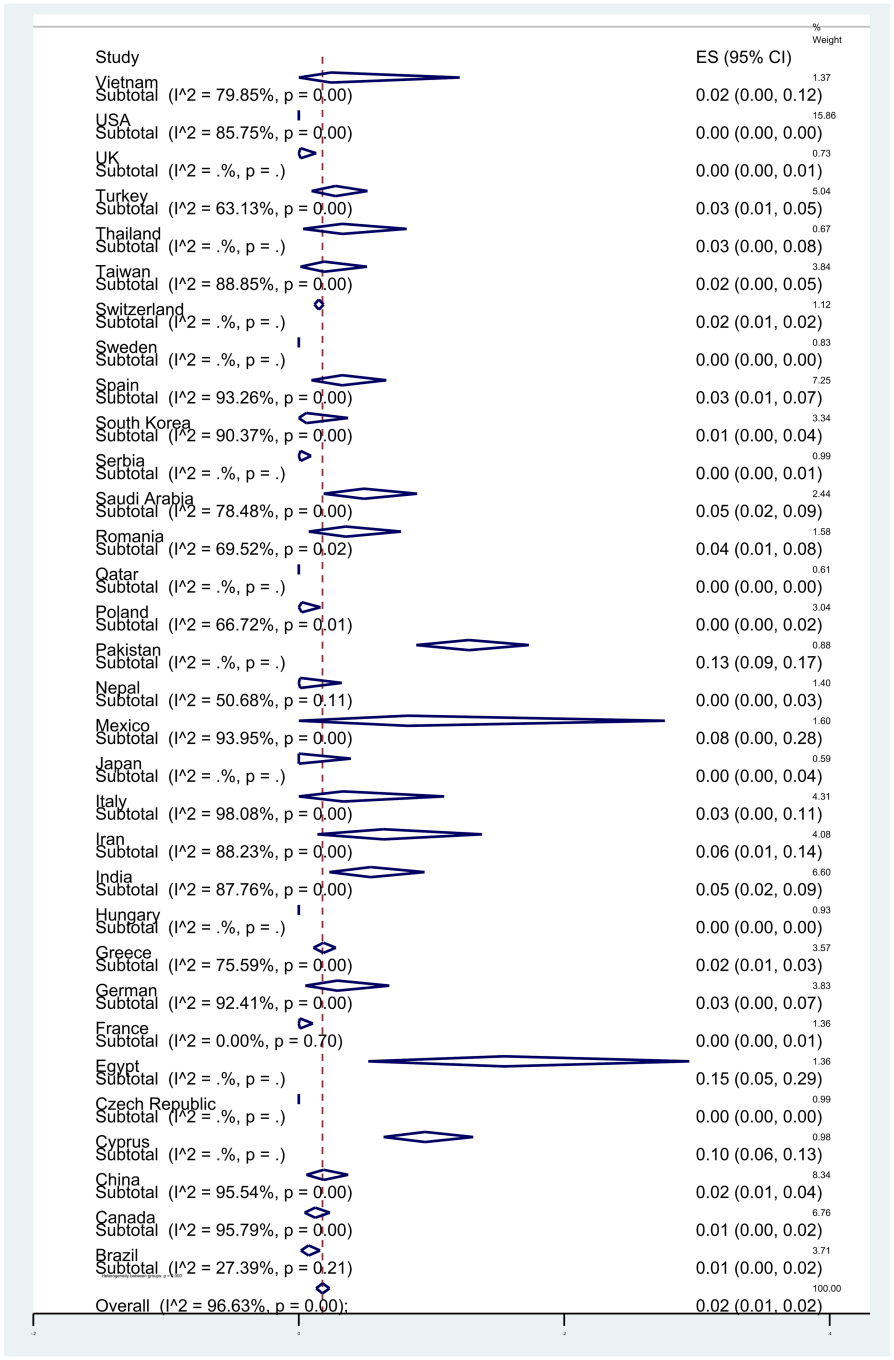
Subgroup analysis for countries of the colistin-resistant *P. aeruginosa* isolates.

Subgroup meta-analyses based on continents indicated that Africa exhibited the highest resistance rate at 4% (95% CI: 0–13%) (*p* < 0.001) (Figure 3). The studies encompassed 32 countries, with Egypt 15% (95% CI: 5–29%), and Pakistan 13% (95% CI: 9–17%) had the highest resistance (*p* < 0.001) (Figure 4).

**Figure 5:**
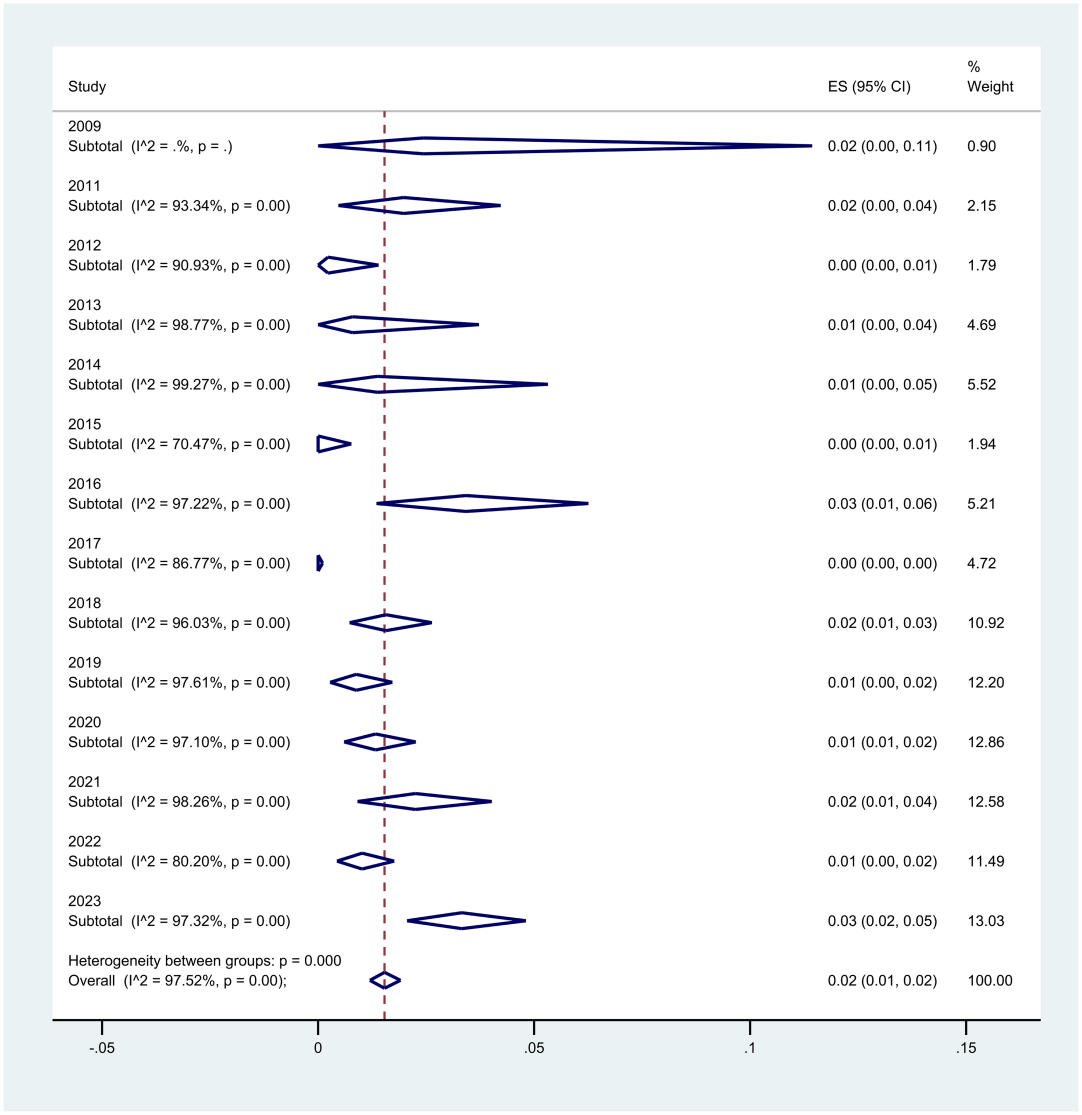
Subgroup analysis for year of publication of the colistin-resistant *P. aeruginosa* isolates.

Regarding the subgroup meta-analyses based on the year of article publication, the rate of *P. aeruginosa* resistance to colistin has increased from 2% (95% CI: 0–11%) in 2009 to 3% (95% CI: 2–5%) in 2023, representing a 1% increase (*p* < 0.001) (Figure 5).

**Figure 6:**
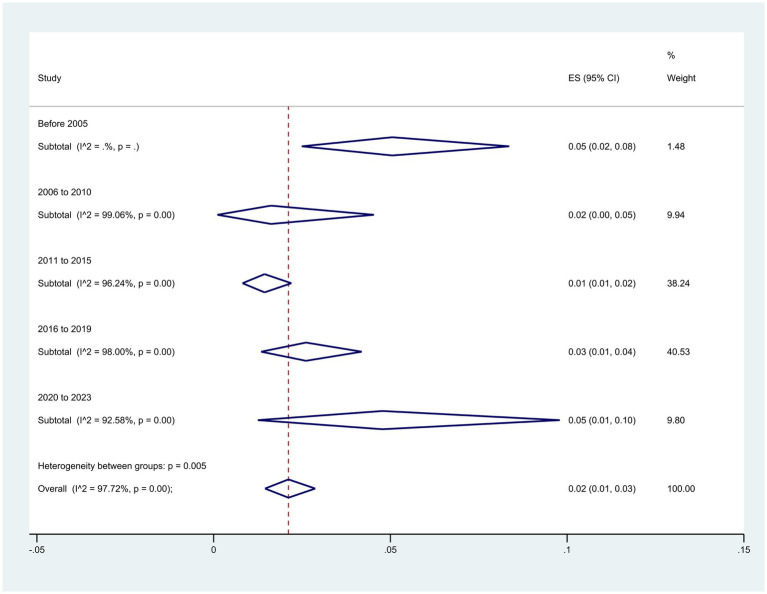
Subgroup analysis for period of sample collection of the colistin-resistant *P. aeruginosa* isolates.

When the sample collection time was divided into five periods, the subgroup meta-analyses revealed an increase in resistance over time from 2% (95% CI: 0–5%) to 5% (95% CI: 0–10%), from 2010–2006 to 2020–2023 (*p* = 0.005) (Figure 6).

**Figure 7:**
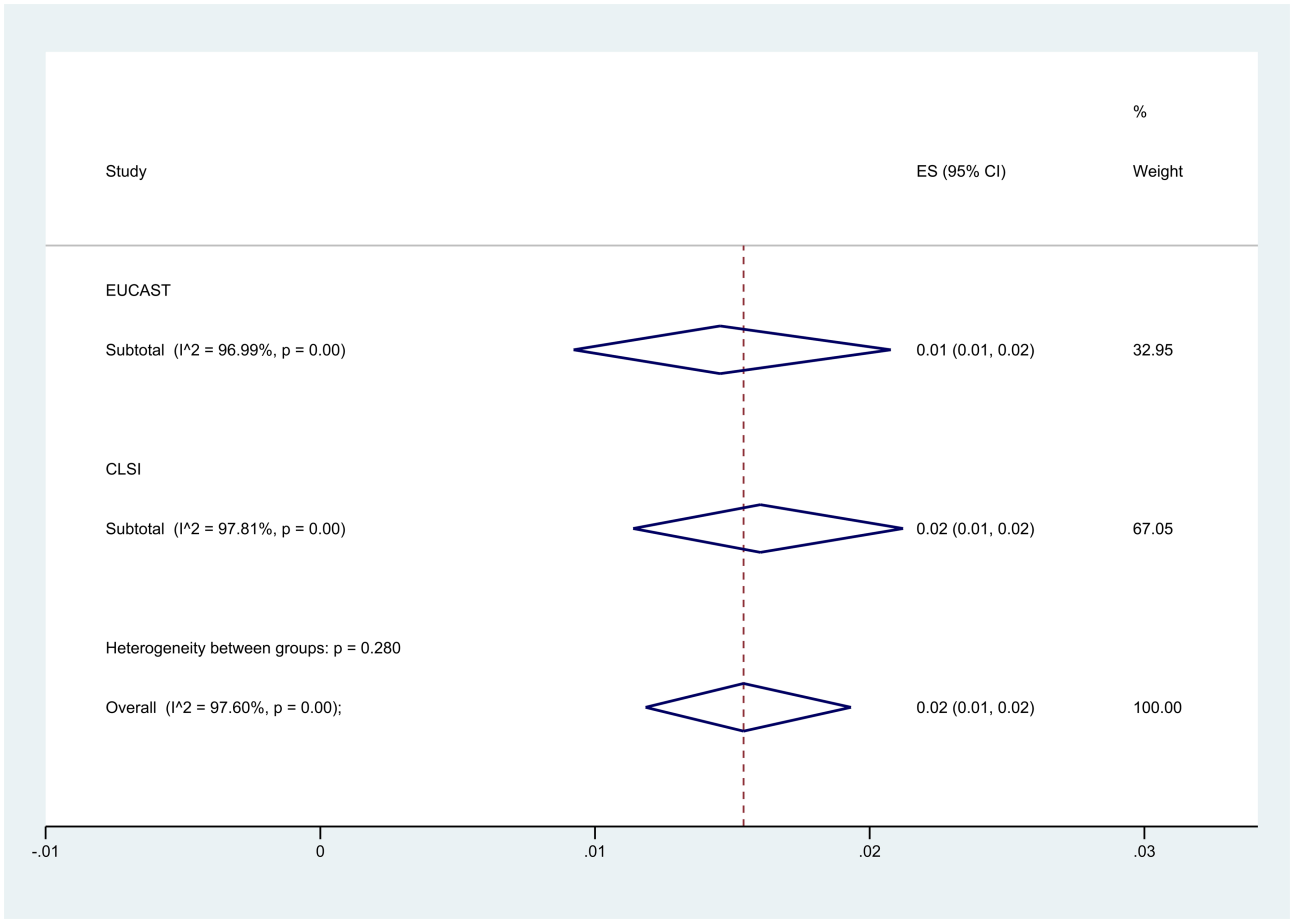
Subgroup meta-analysis for guidelines of colistin-resistant *P. aeruginosa* isolates.

Subgroup analysis based on guidelines demonstrated that the CLSI group had a higher resistance level at 2% (95% CI: 1–2%) compared to 1% (95% CI: 1–2%) in the EUCAST group (*p* = 0.280) (Figure 7).

**Figure 8:**
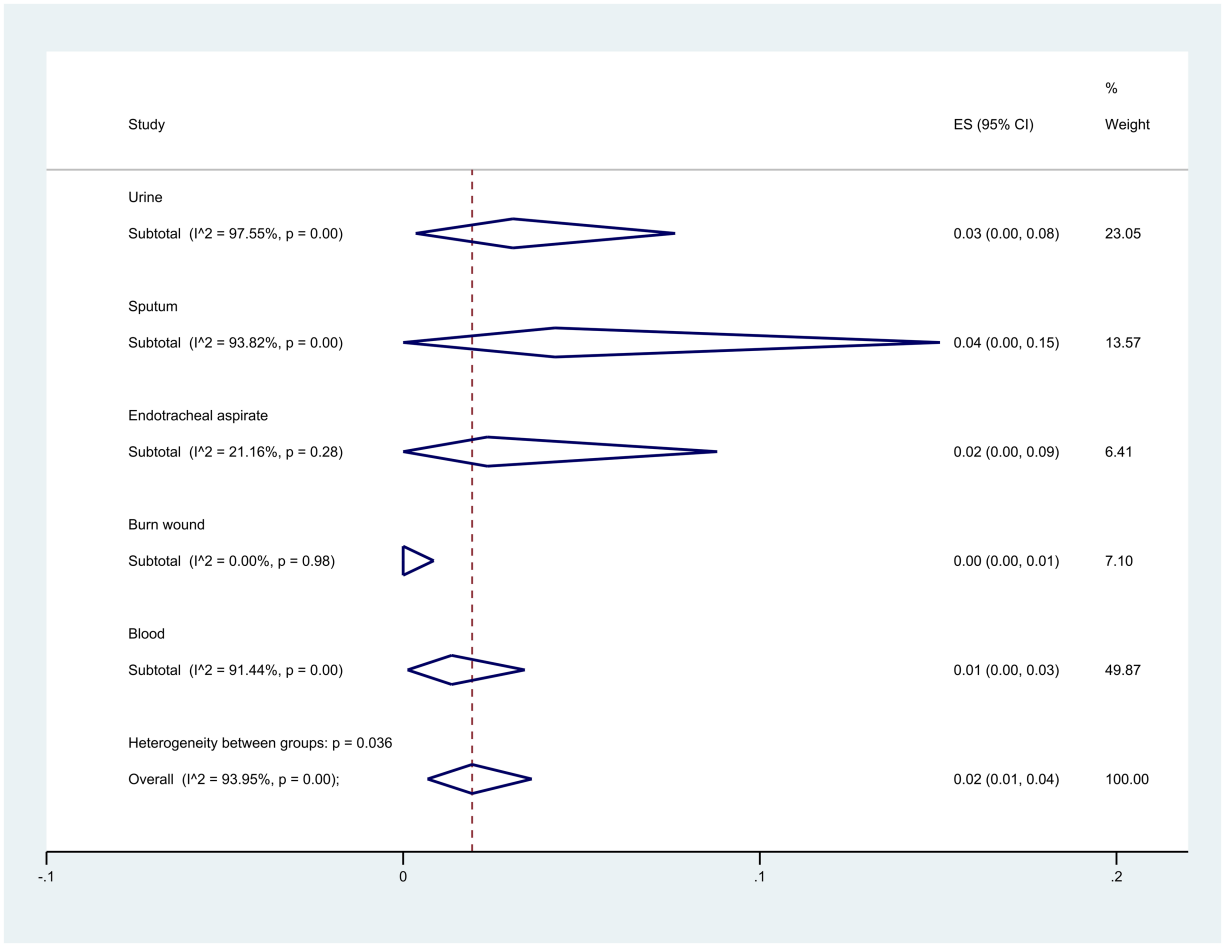
Subgroup analysis for origin of samples of colistin-resistant *P. aeruginosa* isolates.

Regarding the origin of samples, sputum samples exhibited the highest resistance at 4% (95% CI: 0–15%), whereas burn wound samples showed the lowest at 0% (95% CI: 0–1%) (*p* = 0.036) (Figure 8).

**Figure 9:**
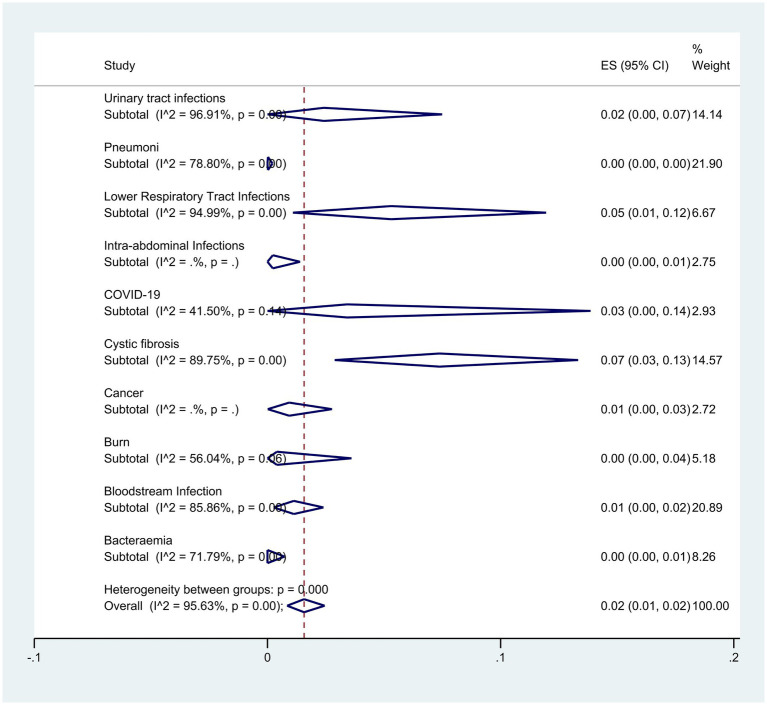
Subgroup analysis for infection of colistin-resistant *P. aeruginosa* isolates.

Subgroup analysis based on disease type showed that patients with cystic fibrosis and lower respiratory infection had the highest resistance with rates of 7% (95% CI: 13–3%) and 5% (95% CI: 1–12%) respectively (*p* < 0.001) (Figure 9).

**Figure 10:**
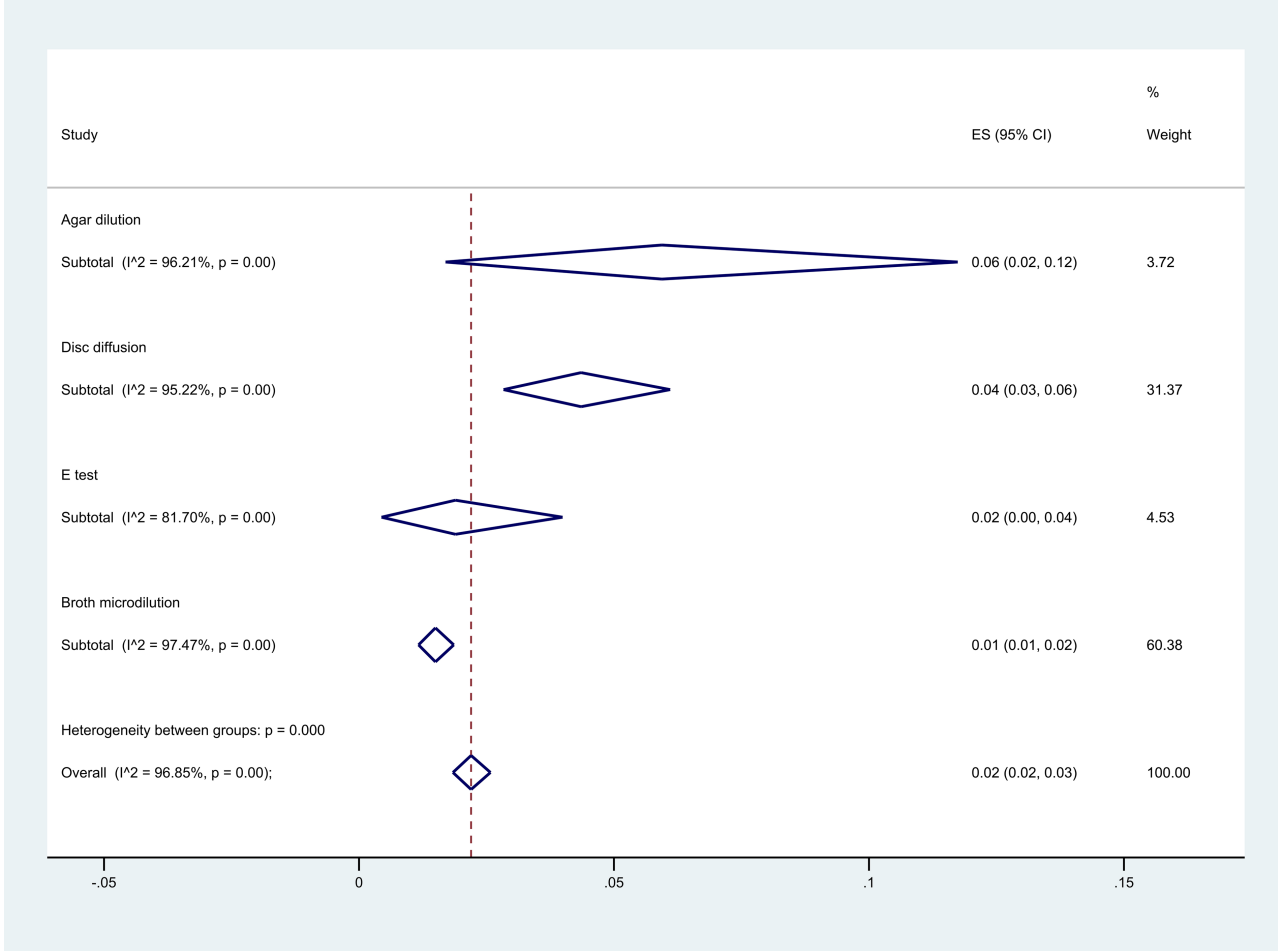
Subgroup meta-analysis for AST methods of colistin-resistant *P. aeruginosa* isolates.

Among the methodologies used, 22 articles employed the agar dilution method, 28 used the E-test method, 192 utilized the disk diffusion method, and 262 opted for the broth microdilution method. The analysis indicated the highest resistance level with the agar dilution method at 6% (95% CI: 2–12%), while the broth microdilution method showed the lowest at 1% (95% CI: 1–2%) (*p* < 0.001) (Figure 10).

**Figure 11:**
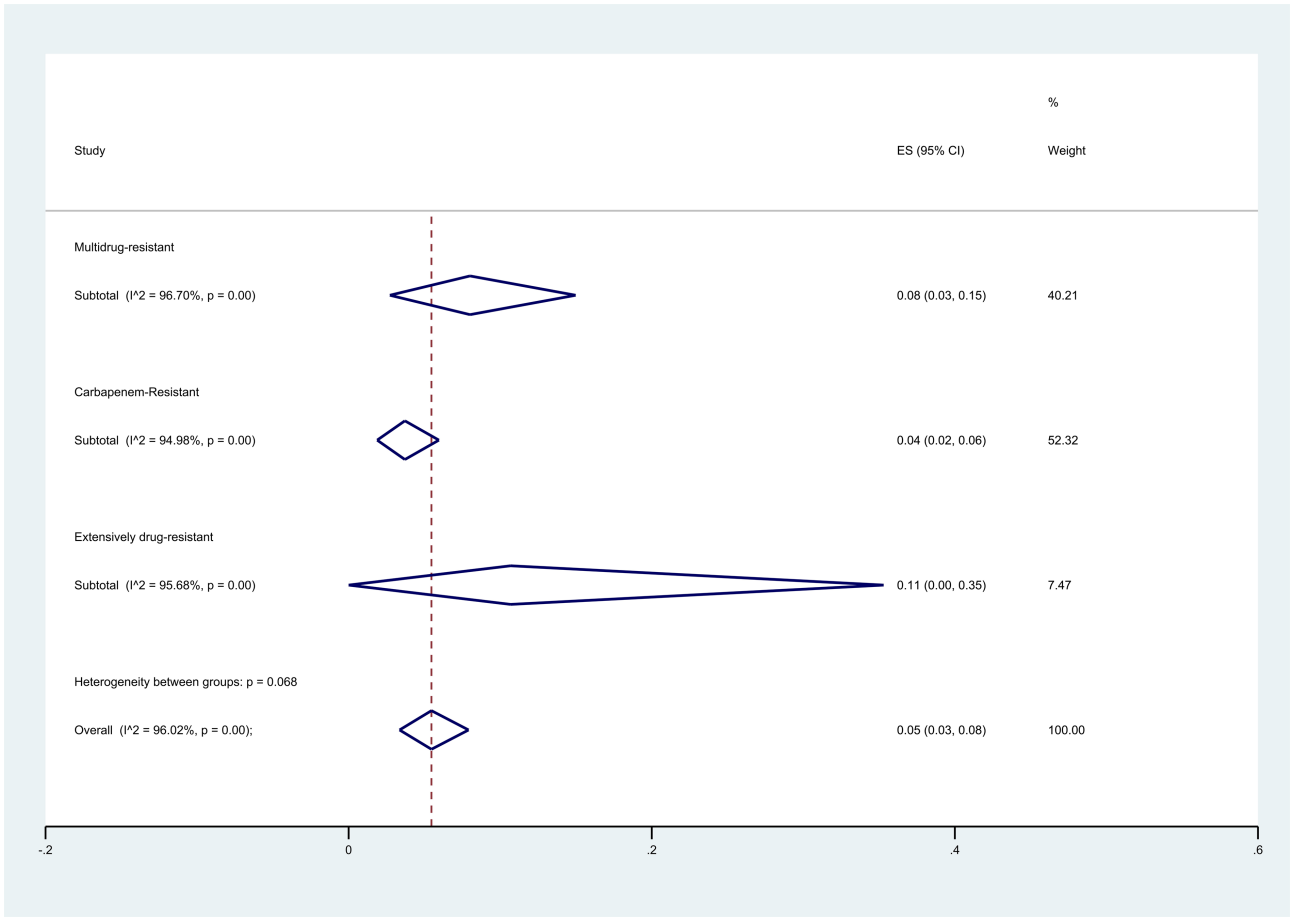
Subgroup meta-analysis for different resistance categories of colistin-resistant *P. aeruginosa* isolates.

When exploring colistin resistance in isolates with different resistance categories, findings based on the broth microdilution and disk elution method revealed that Extensively Drug-Resistant (XDR) isolates displayed a resistance rate of 11% (95% CI: 0–35%), Multidrug-resistant (MDR) isolates displayed a resistance rate of 8% (95% CI: 3–5%), and Carbapenem-Resistant (CR) isolates displayed a resistance rate of 4% (95% CI: 2–6%) (*p* = 0.068) (Figure 11).

## Discussion

Antimicrobial resistance poses a significant threat to public health, leading to increased treatment expenses, prolonged hospital stays, and higher mortality rates. Currently, the rise in antibiotic resistance is particularly concerning in Enterobacteriaceae family members and the hospital bacterium *P. aeruginosa* (Talebi Bezmin Abadi et al., 2019). Additionally, the inappropriate and excessive use of antibiotics in medical and veterinary settings has contributed to the emergence of resistant strains.

*P. aeruginosa* exhibits various intrinsic and acquired antimicrobial resistance mechanisms, including AmpC cephalosporinases, diverse carbapenemases, and multidrug efflux pumps, resulting in resistance to a wide range of antimicrobial agents (Pang et al., 2019). The emergence and dissemination of MDR and XDR strains of *P. aeruginosa*, coupled with the limited availability of effective antimicrobial agents against these bacteria, have severely restricted treatment options (del Barrio-Tofiño et al., 2020; Willmann et al., 2015). Numerous studies have indicated that *P. aeruginosa* is resistant to most beta-lactam antibiotics, quinolones, and aminoglycosides (Pachori et al., 2019). Although carbapenems have been considered one of the primary treatment choices for *P. aeruginosa* infections, increasing resistance to this antibiotic has imposed limitations on its use (Pang et al., 2019; Balkhair et al., 2019). Despite colistin and tigecycline being commonly viewed as the only available antimicrobial agents for treating XDR *P. aeruginosa* infections, some strains have developed resistance to these last-line treatment options (Pang et al., 2019; Ibrahim et al., 2021; Cai et al., 2012). The widespread use of colistin has created conditions conducive to the emergence of resistant strains (Bialvaei and Samadi, 2015). The objective of this meta-analysis was to investigate the global prevalence of colistin resistance in *P. aeruginosa* isolates.

Our findings revealed that the estimated overall prevalence of colistin resistance in clinical isolates of *P. aeruginosa* was 1%. Several other meta-analyses have examined colistin resistance in different Gram-negative bacteria, finding rates of 6.9% in Iran for *Klebsiella pneumoniae* (Narimisa et al., 2022) and 4% in *Acinetobacter baumannii* (Bostanghadiri et al., 2024). While the level of colistin resistance in *P. aeruginosa* has been lower than in other Gram-negative bacteria, our analysis indicates that this resistance has been increasing in recent years. The recent rise in colistin resistance among *P. aeruginosa* can be attributed to multiple factors, particularly the overuse and misuse of antibiotics in both clinical and agricultural contexts, which have intensified selective pressure on bacterial populations (Hassen et al., 2022). Colistin, recognized as a last-resort antibiotic for multidrug-resistant infections, has seen increased utilization due to the emergence of resistant pathogens (Sharma et al., 2022), especially during the COVID-19 pandemic. As healthcare systems faced a surge in respiratory infections, colistin was often employed as a last-resort treatment, further heightening selective pressure on bacteria (de Blasio, 2021). This surge in usage, frequently driven by empirical treatment strategies amid uncertainty, has facilitated the emergence and spread of resistant strains (Ghosh et al., 2021). Additionally, the pandemic disrupted routine healthcare practices and antibiotic stewardship programs, creating an environment conducive to the development of resistance (Campbell et al., 2023). The implications of this growing resistance are significant, complicating treatment options for infections caused by multidrug-resistant organisms and presenting considerable challenges for public health.

Our analysis revealed that the highest rates of colistin resistance were observed in Egypt (15%) and Pakistan (13%). The implementation of effective management strategies is essential for the appropriate use of this antibiotic in these regions. This issue may be attributed to the lack of adequate diagnostic tools, as patient management often relies heavily on drug prescriptions, particularly antibiotics, in developing countries. Additionally, the availability of substandard antibiotics sold over the counter further exacerbates the problem, contributing to the rising rates of antimicrobial resistance in these areas (Dadgostar, 2019; Chaw et al., 2018; Chokshi et al., 2019).

Having a comprehensive standard protocol for determining antibiotic sensitivity is critical for several antibiotics. The CLSI recommends utilizing broth microdilution, colistin broth disc elution (CBDE), and the colistin agar test (CAT) for antimicrobial susceptibility testing against colistin. Additionally, the European Committee on Antimicrobial Susceptibility Testing (EUCAST) also advocates for broth microdilution as the preferred method for evaluating susceptibility to colistin (Pancholi et al., 2018; Özhak et al., 2019). The disk diffusion method, a widely employed and cost-effective approach in clinical microbiology laboratories, particularly in developing countries, lacks a standardized protocol for colistin sensitivity testing (Waites et al., 2011). In a study by Irene Galani and colleagues in Greece, two phenotypic methods, E-test and disk diffusion, were compared for measuring colistin resistance in Gram-negative bacilli. The researchers concluded that the disk diffusion method is not suitable or reliable for assessing antimicrobial sensitivity to colistin (Galani et al., 2008). Despite this, numerous articles have utilized non-endorsed methods, such as disk diffusion, to assess resistance. A subgroup meta-analysis focusing on measurement methods consistently found higher resistance rates when alternative methods were used, compared to the standard method. This discrepancy may be attributed to the lack of sensitivity in other methods for detecting and distinguishing resistant strains. Therefore, adherence to established standard guidelines for measurement methods is imperative.

The findings of our study revealed that *P. aeruginosa* isolated from respiratory samples, particularly in patients with respiratory infections such as cystic fibrosis, exhibited the highest level of resistance to colistin. In a meta-analysis conducted by Bonyadi et al. (2022), the resistance rate of *P. aeruginosa* isolates from cystic fibrosis patients to colistin was reported as 5%. However, our study demonstrated a resistance rate of 7%, suggesting a potential increase in resistance over the past two years. Notably, our subgroup meta-analysis focused solely on studies where resistance rates were confirmed by established guidelines, which may account for the variance in resistance percentages among cystic fibrosis patients.

Given the challenges in discovering new antibiotics, optimizing the use of existing treatments is crucial. Colistin is considered the last resort for treating extensively drug-resistant (XDR) Gram-negative bacteria (Ozsurekci et al., 2016). To address the increasing rates of antibiotic resistance, it is vital to implement innovative strategies. For instance, the combination of colistin and other antibiotics has demonstrated a synergistic effect against antibiotic-resistant Gram-negative pathogens, potentially curtailing the development of resistance (Ly et al., 2015). Other approaches may include combination therapies utilizing nanoparticles, natural components, and phage-based strategies (Holger et al., 2022b; Yassin et al., 2022; Wang et al., 2022). Additionally, promoting antibiotic stewardship and preventing the misuse and overprescription of colistin, particularly among physicians in developing countries, is essential for maintaining its effectiveness.

The considerable heterogeneity among the studies represents a primary limitation of this research. Nonetheless, through the use of subgroup analysis, we were able to identify sources of heterogeneity and mitigate its impact on the outcomes. Another limitation of this article is the exclusion of non-English studies, which may contain valuable data. The decision to focus solely on English-language research aimed to ensure accurate comprehension of the studies and maintain consistency in data quality and reporting. However, we recognize that this exclusion may compromise the comprehensiveness of our analysis. We encourage future research to incorporate studies in other languages to provide a more comprehensive view of the topic.

## Conclusion

Our study indicates that while the overall rate of resistance to colistin in *P. aeruginosa* is relatively low, there has been a recent upward trend in resistance levels. This underscores the importance of accurate surveillance of resistance rates, particularly in regions with higher prevalence, and the judicious prescription of antibiotics for patients with *P. aeruginosa* infection. Promoting antibiotic stewardship and preventing the misuse and overprescription of colistin, especially among healthcare professionals in developing countries, is crucial for preserving its efficacy.

## Data Availability

The original contributions presented in the study are included in the article/Supplementary material, further inquiries can be directed to the corresponding author.
